# Marginal Microleakage of Low-shrinkage Composite Silorane in Primary Teeth: An In Vitro Study

**DOI:** 10.5681/joddd.2012.020

**Published:** 2012-09-01

**Authors:** Hamid Reza Poureslami, Fatemeh Sajadi, Maryam Sharifi, Shahram Farzin Ebrahimi

**Affiliations:** ^1^Oral & Dental Diseases Research Center, Kerman University of Medical Sciences, Kerman, Iran; ^2^Associate Professor, Department of Pediatric Dentistry, Dental School, Kerman University of Medical Sciences, Kerman, Iran; ^3^Assistant Professor, Department of Pediatric Dentistry, Dental School, Kerman University of Medical Sciences, Kerman, Iran; ^4^Assistant Professor, Department of Pediatric Dentistry, Dental School, Rafsanjan University of Medical Sciences, Rafsanjan, Iran; ^5^Assistant Professor, Department of Oprative Dentistry, Dental School, Kerman University of Medical Sciences, Kerman, Iran

**Keywords:** Microleakage, polymerization, shrinkage

## Abstract

**Background and aims:**

Despite the increasing demand for adhesive restorations in pediatric dentistry, polymerization shrinkage and subsequent marginal microleakage remains a problem. The aim of this study was to evaluate of the sealing ability of novel low-shrinkage composite silorane in class V cavity of primary canines in comparison with three types of composite resin.

**Materials and methods:**

Ninety-one non-carious extracted primary canines were randomly divided in six groups (n=15). Standard class V cavities were prepared on the buccal surface of each tooth that the occlusal margin was in the enamel and the cervical margin extending 1 mm below the cemento-enamel junction. The preparations were restored with the different composite materials in normal consistency with application the bonding in six groups (Filtek silorane; etch + Filtek Silorane; Z250; Filtek supreme; els saremco; Aelite LS). Teeth were then exposed to thermal cycles (1000 cycles, 5°C and 55°C), sealed and immersed in a 0.5% basic fuchsine for 24 hours, and finally sectioned. Buccolingual and mar-ginal leakage was assessed with dye penetration.

**Results:**

The best seal were obtained with etch + Filtek Silorane (P < 0.05) and the weakest seal with Z250 composite res-torations (P > 0.05). Except for etch + silorane, there was no significant differences in sealing ability (P > 0.05), and in the microleakage degree at the cementum and enamel margins (P > 0.05) between the groups.

**Conclusion:**

According to the results, low-shrinkage silorane composite restorations with etching the cavity provide the highest seal in primary teeth.

## Introduction

There is an increased demand for esthetic restorations in pediatric dentistry.^1^ Resin composite materials have improved greatly since their introduction. Although composites are used widely, polymerization shrinkage remains a problem with composite restorations that leads to inadequate adaptation with the cavity wall and microleakage.^2 , 3^

Polymerization shrinkage is mainly dependent on chemical composition of the resin matrix. As a result, researchers have introduced changes in matrix composition such as replacing the linear methacrylate monomer with non methacrylate ring-opening monomers in order to minimize the polymerization shrinkage.^2 , 4^

Although knowledge of filler shape and composition are still important, the development of various matrix components necessitates an additional material classification: (1) Conventional matrix (methacrylate base): hybrid and nano-composite; (2) Ring opening epoxide: silorane; (3) Inorganic matrix: ormocer; (4) Acid modified methacrylate: compomer.^5^

Silorane is ring-opening, silicon based monomer which is composed of siloxan and oxirane. The ring opening character of monomer reduces polymerization shrinkage below 1% volumetric.^6 , 7^ Several studies have been performed on this composite and its clinical properties. Palin et al^8^ showed that microleakage was significantly lower for a silorane material than other composites (Z250, 3M ESPE). Al-Boni et al^3^ also showed that low shrinkage silorane system had lesser microleakage than other methacrylate resin based composite (Amelogen, Filtek Z250). In another study, Krifka et al^9^ found that Filtek Silorane, a silorane-based composite resin, exhibits the best marginal seal in comparison with methacrylate-based composite. The three-step adhesive yielded better marginal sealing compared with the one-step adhesive for methacrylate-based class V composite restorations.

The objective of this study was to compare the sealing ability of silorane material with four different types of other methacrylate composites in class V cavity restorations in primary teeth in vitro.

## Materials and Methods

Ninety sound human primary canines extracted for orthodontic reasons were examined. The teeth had no fracture, crack or pigmentation with at least 10 mm of root structure remaining. As for using extracted teeth, there were no special considerations from Research Ethics Committee. Samples were stored in physiologic saline at 37°C and restored maximum one month after extraction.^10^

The teeth were randomly divided to 6 groups with 15 teeth in each group. Standardized class V cavity (3 mm × 2 mm × 2 mm) was prepared at the buccal surface of each tooth (diamond fissure 330; S.S White, Washington, USA). The occlusal margins of cavities were prepared at the enamel and gingival margin extending 1 mm below the cementoenamel junction (CEJ). Each bur was used for five preparations. Then, each group was restored with one type of composite in normal consistency with the application of bonding according to Table 1. In all groups, the cavities were restored in three increments: the first layer on the axial wall, the second layer extending from occlusal wall to the gingival, and the third layer extended from gingival to occlusal; so that the layers overlapped.^11^ Polymerization of the each layer was done separately (1000 mW/cm²; Coltolux 75, Coltene, USA). The restorations were finished (finishing bur No 820621; Teezkavan, Tehran, Iran) and polished (flexible disk, 80-3µm, Softlex XT pop-on, 3M ESPE, USA) under simultaneous water cooling.

**Table 1:** The restorations used in each study group

Groups	Restoration
Group 1	Bonding Silorane + Silorane (3M/ESPE, St. Paul, USA)
Group 2	Etch + Bonding Silorane + Silorane (3M/ESPE, St. Paul, USA)
Group 3	Bonding (T Adper Scotch Bond Self-Etch Adhesive) + Z250 composite (3M/ESPE, St. Paul, USA)
Group 4	Bonding (T Adper Scotch Bond Self-Etch Adhesive) + Filtek supreme (3M/ESPE, St. Paul, USA)
Group 5	Etch microcid +Bonding (James-2) + Saremco LS (Saremco, St. Gallen, Switzerland)
Group 6	Uni-Etch (32%) W/BAC + Bonding (one step plus OS+) + Aelite LS Bisco (Bisco Inc., Schaumburg, USA)

The teeth were thermocycled under 5–55°C (1000 cycles, 60 second for each cycle).^12^ Then, the specimens were dried, the apex was sealed with light-cured composite, all the teeth surfaces were sealed with two layers of waterproof varnish except 1 mm around the restoration margins and immersed in 0.5% basic fuchsine dye at room temperature.^13^

After 24 hours, the teeth were retrieved from dye solution, washed, dried and mounted in acrylic resin. Then, the specimens were sectioned with diamond disk in labiolingual direction and from the center of the restoration. The samples were examined under a stereomicroscope with ×40 magnification (Kyowa optical, SDZ-TR-PL, Japan). For examination of microleakage, the Kappa index was 80%. The degree of microleakage was evaluated and scored as follows: 0: No dye penetration; 1: Dye penetration along the incisal or gingival wall less than the total length of the wall; 2: Dye penetration along the entire length of the incisal or gingival wall; 3: Dye penetration along the entire length of the incisal or gingival wall as well as the axial wall.^1^

All data were analyzed using U Mann-Whitney test to determine the significant differences between groups. The level of significance have considered at p value <0.05.

## Results

The microleakage scores in enamel and cementum margins of the six groups are shown in Table 2. The best seal was obtained in group 2 (Filtek Silorane + etch) (P < 0.05), followed by group 4 (Supreme), group 5, (els Saremco), group 6 (Aelite LS), and group 1 (Filtek Silorane), respectively. The weakest seal was obtained in group 3 (Z250) (P > 0.05). Except for group 2 (Siloranev + etch), there were no statistically significant differences in microleakage scores between the other groups, according to U Mann-Whitney test. Also, no significant statistically differences were recorded in the microleakage scores between the cementum and enamel margins.

**Table 2:** Frequency of marginal microleakage scores in enamel and cementum in the study groups

	Group 1	Group 2	Group 3	Group 4	Group 5	Group 6
Margin	0 1 2 3	0 1 2 3	0 1 2 3	0 1 2 3	0 1 2 3	0 1 2 3
Enamel	2 3 2 9	12 3 0 0	2 0 6 7	4 7 0 4	3 6 3 3	1 5 3 6
Cementum	1 3 3 9	11 4 0 0	0 5 3 7	4 3 2 6	6 2 2 5	3 2 4 6

Group 1: Bonding Silorane + Silorane; Group 2: Etch + Bonding Silorane + Silorane; Group 3: Bonding + Z250 composite; Group 4: Bonding + Filtek Supreme; Group 5: Etch Microcid + Bonding + Saremco LS; Group 6: Uni Etch W/BAC 32% BISCO+ Bonding + Aelite LS Bisco. Microleakage scores: 0: No dye penetration; 1: Dye penetration along the incisal or gingival wall less than the total length of the wall; 2: Dye penetration along the entire length of the incisal or gingival wall; 3: Dye penetration along the entire length of the incisal or gingival wall as well as the axial wall.

## Discussion

Prevention of microleakage in composite restorations is the main purpose in operative dentistry. Microleakage caused by the gap in the interface of restoration and tooth structure may lead to recurrent caries and pulp inflammation.^2^ One of the measures for assessing the marginal adaptation of the composite restorations is microleakage,^14^ and dye penetration is the most common technique for assessing the microleakage.^15^ In this study basic fuchsine dye was selected due to its easy manipulation,^16^ and Class V cavities were selected because they do not have any macro-mechanical undercut, so that the sealing ability of composite restorations were evaluated just based on the bonding effect.^17^

According to our results, the best marginal seal was observed in silorane composite restorations with application of acid etch and there were no significant difference between the other groups in the scores of microleakage.

Silorane composite is a novel low-shrinkage composite which contain silorane monomers with ring-opening ability in resin matrix instead of traditional methacrylate monomers.

Laboratory studies have indicated that polymerization shrinkage of silorane composite is below 1% volumetric.^6 , 7^ Since silorane matrix is highly hydrophobic, it requires an individual bonding system called silorane system adhesive (SSA). SSA is a two-step self-etch adhesive but bonding to dental structures is obtained in the first application step similar to one-step self-etch adhesives. The self-etch primer in the SSA cannot dissolve the smear layer within the tubules due to its pH of 2.7 and is classified as ultra mild etchant.^18^

Recent studies have shown no significant statistical difference in marginal microleakage between the restorations with silorane + SSA and other metacrylate composites (Z250), because in silorane restorations, there is no efficient and effective bond at the interface of prepared tooth structure and SSA primer despite low polymerization shrinkage of Silorane matrix.^19 , 20^

In silorane + etch restorations smear layer and smear plug was removed by using 37% phosphoric acid before application of SSA. This reveals that silorane could reduce microleakage if sufficient and efficient bond were obtained between the composite and tooth interface. The maximum marginal microleakage was observed in Z250 composite with two-step self-etch bonding. Studies have indicated that there is no significant differences between two-step self-etch and two-step full-etch bondings.^3 - 19^So, polymerization shrinkage is the main reason for microleakage in this composite.

Following groups 2 & 3 in the present study, the maximum marginal microleakage was observed in Bisco restorations, which have high filer composition, the cause of their high coefficient of elasticity and low flowability. So, these factors are the reasons for high microleakage in this group.

This study showed that there was no statistically significant difference in microleakage of SAREMCO group with other groups except for group 2 (silorane + etch). HEMA and TEGDMA omitted from bonding of this composite that the studies have shown there are no different in bonding strength in this bonding system with two steps methacrylate bonding agents.^21^

Also, in this study there were no statistical significant microleakage at apical and coronal margins, which is in line with previous findings.^11 , 12 , 14 , 2^ The reason for this finding is related to enamel and dentin thicknesses. The enamel thickness in primary teeth is less than that in permanent teeth and enamel thickness in cervical area is lower than the coronal margin. Therefore, in class V restorations, the maximum bond is with dentin and there are no differences in microleakage of apical and coronal.^14^

## Conclusion

This study demonstrated that etching the cavity before application of silorane composite could increase the bonding efficiency in primary teeth and silorane restorations can provide an acceptable marginal seal due to its low level of polymerization shrinkage. However, more in vitro and clinical studies are needed to confirm these results.
